# The role of graft cross-linking during keratoplasty in patients with corneal melting

**DOI:** 10.1038/s41598-024-66629-2

**Published:** 2024-07-11

**Authors:** Raphael Kilian, Gerald Schmidinger, Jan Lammer

**Affiliations:** 1 Department of Translational Medicine, University of Ferrara, Ferrara, Italy; 2 Department of Ophthalmology and Optometry, Medical University of Vienna, Waehringer Guertel 18-20, 1090 Vienna, Austria

**Keywords:** Corneal diseases, Scleral diseases

## Abstract

The purpose of this study was to investigate the role of corneal crosslinking (CXL) of grafts during keratoplasty (KP) in patients with refractory corneal melting (CM). This is a retrospective case series reporting the clinical outcomes of patients who received a crosslinked corneal graft during penetrating or deep anterior lamellar KP for refractory infectious or sterile CMs. Outcome measures were the recurrence of CM, the time required for epithelial healing following KP, incidence of complications, and necessity for re-transplantation. Twenty eyes of 18 patients with a follow-up of 29.2 ± 15.8 months were included in this study. All but two eyes had undergone previous KPs during the course of their disease (mean 1.9 ± 1.6). After CXL-enhanced KP, three eyes (15%) experienced recurrence of CM, three eyes developed an infectious keratitis and six eyes (30%) required a re-transplantation (three of them within 12 months). The mean time to epithelium closure after CXL-enhanced KP was 63 ± 90 days. The number of postoperative re-transplantations was significantly lower than the number of KPs performed before the CXL-enhanced transplantation (before CXL 1.9 ± 1.6 vs after CXL: 0.3 ± 0.57, p = 0.002). To conclude, CXL of the graft at the time of keratoplasty decreased the need for re-transplantations. However, further studies are needed in order to establish its role in the management of severe CM necessitating therapeutic corneal transplantation.

## Introduction

Corneal melting (CM), or keratolysis, is one of the most serious complications of many infectious and non-infectious corneal disorders. Its management consists in the treatment of the underlying condition (e.g., antibiotic/fungal/viral agents in cases of infectious keratitis [IK]), of the persistent epithelial defect (PED; e.g., lubricating eye drops, amniotic membrane, autologous serum), and of the melt itself, and might include various systemic or topical metalloproteinase inhibitors (e.g., oral doxycycline or topical cysteine). Despite a correct initial therapeutic strategy, CM can still result in the development of corneal perforation and possibly endophthalmitis^1^. To avoid these complications, in the latest stages of the disease, an “a chaud” (a.k.a. acute therapeutic) keratoplasty (KP) is often performed. However, these fail in up to 50% of eyes with both infectious or non-infectious keratitis, the major causes of failure being infection- and/or CM-recurrence associated with PEDs^2,3^.

In the last decade, corneal crosslinking (CXL), a technique that has become popular for the management of corneal ectatic disorders, has been increasingly proposed in eyes with infectious and sterile CMs^4^. Particularly, this treatment seems to help the corneal stroma to resist proteolysis induced by the enzymes secreted by polymorphonuclear leukocytes during inflammatory reactions, therefore halting the primary process of CM^5^. Despite there being evidence supporting the use of CXL in patients with active inflammatory or infective corneal meltings, there is still much debate on the precise role and the effectiveness of this technique. Indeed, there have also been reports where CXL itself was identified as the cause of the melt^6^.

To help understand the impact of CXL of grafts on the recurrence rate of CM after keratoplasty, we analyzed the outcomes of eyes with recurrent and refractory keratolysis undergoing a transplantation with a crosslinked (CXL-enhanced) graft.

## Methods

In this retrospective case series, we evaluated the clinical records of patients with recurrent and refractory CM who underwent keratoplasty (i.e., either penetrating keratoplasty [PKP] or deep anterior lamellar keratoplasty [DALK]), receiving a graft that was crosslinked during the same procedure (CXL-enhanced KP). All patients underwent surgery at the Allgemeines Krankenhaus der Stadt Wien, Austria, from January 1, 2016, to December 31, 2022, and were transplanted due to refractory CM with or without an actual or impending corneal perforation. All investigations adhered to the tenets of the Declaration of Helsinki and the study was approved by the Institutional Ethics Committee of the Medical University of Vienna (EK-Nr.: 1611/2023). All included patients signed an informed consent for participation in the study.

Corneal melting was defined as a progressive stromal dissolution with an overlying disrupted corneal epithelium, and could be identified as being able to lift strands of loose sticky corneal tissue with a cotton swab^7^. The melt was considered sterile or infectious based on the results of corneal cultures and polymerase chain reactions (PCR), and was defined as refractory when it did not improve within 2 weeks of conservative management.

After evaluating the status of the endothelium and the extension of the melt at the slit lamp and on anterior segment optical coherence tomography (OCT, Casia 2, Tomey GmbH, Germany), the surgeon chose whether to perform DALK or PKP, and the size (diameter) of the transplant. All procedures were performed under general anesthesia and included CXL of the donor graft before its actual implantation. This was done on a separate operating table than the one used for keratoplasty, minutes before the actual transplantation and included the following steps as published previously^8^: (1) the graft was positioned on an artificial anterior chamber, (2) the epithelium was mechanically debrided, (3) riboflavin eye drops (Vibex Rapid, Simovision BV, Belgium) were applied to the graft’s surface for 10 min (i.e., one drop every 1–2 min), and (4) the graft was then irradiated with UV-A light (370 nm wavelength) for another 10 min (irradiance of 9 mW/cm^2^, accumulated irradiance of 5.4 mJ/cm_2_). Finally, the cornea was trephined and transplanted into the recipient following standard-of-care procedures for either the DALK or PKP procedure. Postoperative treatment consisted of the administration of topical corticosteroids (started q.2.h. for 2 weeks and then tapered one drop every 2 weeks until one drop per day, which was maintained indefinitely), antibiotics (q.i.d., maintained until the epithelial defect was healed), and lubricating eye drops (administered up to every hour). All eye drops were administered using a preservation-free formula.

During the retrospective acquisition of data, we looked for and reported any past or concomitant conditions that could have had a negative influence on the results of the surgery (i.e., risk factors for transplant failure), the precise indication for surgery, the number and types of previous transplantations in the same eye, the amount of time the corneal epithelium had been disrupted before surgery, the time interval between the last transplantation and the current procedure, the number and types of postoperative complications, the time at which these complications occurred, their treatment, the need for repeated keratoplasty (re-KP), and the time needed for the epithelium to heal after CXL-enhanced surgery. Graft failure was defined by the need for retransplantation or in cases where final visual acuity was hand motion or less due to graft opacity.

The results of descriptive analyses were expressed as means and standard deviations (SD), medians, or percentages. Statistical differences were evaluated using the Wilcoxon signed rank-test, and a p-value ≤ 0.05 was considered statistically significant.

## Results

Twenty eyes of 18 patients (nine females and nine men) with a mean age of 59 ± 18 years were included in this retrospective analysis. The pre- and postoperative data are summarized in Table 1. Notably, all but two eyes had received previous keratoplasties prior to the CXL-enhanced KP (mean 1.9 ± 1.6) and seven eyes (35%) had at least one previous failed amniotic membrane transplantation. The median time between the previous KP and CXL-enhanced KP, was 7.5 months (range 1–144 months, mean 30.5 ± 47.5 months).

**Table 1:** Pre- and post-operative characteristics of included eyes.

	1	2	3	4	5	6	7	8	9	10	11	12	13	14	15	16	17	18	19	20
Concomitant/past relevant conditions	NK	NK	NK	Pemphigoid	Chemical burn	DM	NK	TEN	TEN	GVHD	TEN	Chemical burn	Atopic dermatitis	Thermal burn	Chemical burn	Past Acanth. IK	Exposure keratopathy	Ocular trauma	Chemical burn	Ectodermal dysplasia
Indication for TX on CM	PED	PED	Perforation	PED	Perforation	TX failure	Perforation	IK on PED	Perforation	IK	Perforation	PED	IK	PED	Perforation	PED	TX rejection	PED	Perforation	Impending perforarion
N and type of previous TX	DALK x1	0	PKP x2	PKP x1	DALK x2	PKP x1	PKP x2	DALK x1	DALK x2 PKP x2	PKP x1	DALKx1, PKP x4	DALK x2 PKP x4	PKP x2	PKP x1	0	PKP x1	PKP x1	DSAEK x1, DMEK x1, PKP x2	PKP x1	DSAEK+AMT x1
T last-current TX (months)	3	/	3	7	120	144	12	8	70	7	5	144	47	15	/	8	1	5	9	3
Duration of preop. Epithelial defect (days)	42	14	82	200	41	14	14	84	84	102	126	1095	43	259	54	124	14	14	277	22
Additional procedures performed on the day of TX	/	/	/	AMT	/	/	/	/	/	/	AMT	/	/	/	/	/	Tarsorraphy	/	/	MT
Follow-up	54	31	55	31	3	12	28	47	46	7	38	46	6	36	39	34	15	21	22	13
Duration of postop. epithelial defect (days)	6	3	7	23	90	7	4	13	13	10	210	151	180	6	330	29	60	7	10	90
Postop. complications	Glaucoma	PED with infection	Infection with CM	None	PED, Glaucoma	None	Glaucoma, Suture loosening	Rejection	PED	PED, Glaucoma	PED, CM	PED, Suture loosening	PED, infection	PED, Glaucoma, Rejection	PED	Glaucom	CM	RD	Infection	PED, CM
Onset of complication after TX (months)	6	11	12	/	0, 6	/	3, 2	8	4	1, 2	0.5, 7	0, 3.5	0	1.5, 7, 18	0	0.3	2	5	5	0.5, 3
N of re-transplantations	0	1	0	0	0	0	0	2	1	0	1	0	0	0	1	0	0	0	0	1
Time to re-transplantation (months)	/	32	/	/	/	/	/	8	22	/	7.5	/	/	/	28	/	/	/	/	3

*AMT* amniotic membrane transplantation, *CB* chemical burn, *CM* corneal melting, *CN* cranial nerve, *DALK* deep anterior lamellar keratoplasty, *DM* diabetes mellitus, *DMEK* descemet membrane endothelial keratoplasty, *DSAEK* descemet stripping automated endothelial keratoplasty, *GVHD* graft versus host disease, *IK* infectious keratitis, *KP* keratoplasty, *LSCD* limbal stem cell deficiency, *N* number, *NK* neurotrophic keratitis, *PED* persistent epithelial defect, *PKP* penetrating keratoplasty, *re-KP* repeated keratoplasty, RD retinal detachment, *T* time, *TB* thermal burn, *TEN* toxic epidermal necrolysis.

The most frequent initial causes of corneal melting were severe chronic ocular surface disorders with subsequent partial limbal stem cell deficiency (i.e., status post chemical/thermal burn, ocular pemphigoid, toxic epidermal necrolysis, graft versus host disease)—10 cases (50%), and long standing neurotrophic corneal disorders (four cases originating from 5th cranial nerve palsies and one case originating after a previous KP in a patient with diabetes)—five cases (25%). Only three eyes (15%) had an active corneal infection at the time of CXL-enhanced transplantation (one case of *Staphilococcus aureus* and two cases of *Candida spp*).

All eyes had had CM for at least 2 weeks before CXL-enhanced corneal transplantation was proposed and all but three eyes (case n. 6, 13, 14) received the previous non-CXL-KP because of a CM.

The median duration of preoperative corneal epithelial defect was 68 days (range 14–1095 days, mean 135 ± 239 days) and 8 eyes (40%) were treated for an actual or impending CM-associated perforation.

The mean duration of follow-up after CXL-enhanced KP was 29.2 ± 15.8 months (range 3–55 months, median 31 months) and the median postoperative duration of the epithelial defect was 13 days (mean 62.45 ± 89.5 days, range 3–330 days). Although this value was less than half the preoperative value (i.e., time of preoperative epithelial defect), the difference was not statistically significant (preoperative median, 68 days vs. pposoperative median, 13 days; p = 0.15). Three eyes (15%) received amniotic membrane transplantation (AMT), and one eye (5%) received tarsorrhaphy.

Three eyes (15%) experienced recurrence of CM, and three eyes (15%) experienced an infection. Among the postoperative CMs, one was caused by an infection, whereas the others were sterile following a new-onset PED after initial epithelial closure. Among the infections, only one was caused by the same pathogen that caused failure of the previous transplant (i.e., *Acanthamoeba*), whereas the others were new-onset infections in the course of the follow-up period. Finally, five eyes (25%) had recurrent PED without CM or infection.

Of the eyes experiencing recurrent PED or CM (N = 12), seven were successfully treated conservatively via topical lubricants, antibiotics and bandage contact lens application, whereas five needed a retransplantation.

None of the included eyes developed sclerokeratitis. Only three eyes (15%) required re-transplantation within 12 months after CXL-enhanced KP due to graft failure, whereas three more eyes were retransplanted after 1 year (1 therapeutic KP and two due to PED-associated scarring).

When comparing the number of all keratoplasties performed prior to the CXL-enhanced KP and that of retransplantations following the procedure during the follow-up period using the Wilcoxon signed-rank test (data did not follow normal distribution), the latter was significantly lower than the former (number of previous KPs: 1.9 ± 1.6 vs number of re-KPs after CXL-KP: 0.3 ± 0.57; p = 0.0016).

## Discussion

Kymionis et al. successfully implanted cross-linked corneal grafts in a few cases of scleral melting and conjunctival dehiscence, obtaining a good tectonic reconstruction of the anterior ocular surface with no postoperative complications^9^. Also, there are reports in favor of the use of CXL on corneal grafts in cases of Boston keratoprostheses with an actual or possible future corneal melting^10^, and in a review by Robert et al., the authors suggested the possible use of cross-linked grafts in patients with recurrent ulcerative keratitis requiring tectonic or conventional keratoplasties^11^. Furthermore, Lammer et al. have previously investigated endothelial cell integrity in an ex vivo study of crosslinked human corneal grafts, showing good safety and no significant loss after the procedure^8^.

In the present retrospective analysis, we significantly reduced the need for re-KPs during the follow-up period (p = 0.0016) by implanting CXL-enhanced corneal grafts in patients with refractory CM. In addition, despite most patients displaying a partial limbal stem cell deficiency or a neurotrophic keratopathy prior to transplantation, the median duration of postoperative epithelial defect was less than half of the preoperative value. The wide range of postoperative epithelial closure-times across included eyes may be due to several factors. As previously specified, four eyes underwent AMT or tarsorrhaphy at the end of the CXL-enhanced KP. These procedures clouded the duration of the postoperative epithelial defect, since epithelial closure could not be evaluated until removal of the AMT-fixating bandage contact lens or removal of the tarsorrhaphy. Furthermore, four out of six eyes with PED lasting more than 30 days after CXL-enhanced KP had previously received two or more KPs, indicating very severe previous ocular histories. The rest of the eyes, however, displayed relatively short epithelial healing times and 25% of eyes developed a postoperative PED without CM or infection. We suggest these results may be related to the lower immunogenicity of the crosslinked graft, causing less inflammatory damage to epithelial cells. However, this must be determined in studies that are specifically designed for this purpose.

Indeed, epithelial defects are a common complication following therapeutic keratoplasties, even without crosslinked grafts. Of notice, in a study on 594 patients with penetrating KP a chaud, Wan et al. have shown a 19% incidence of PEDs, the major risk factors being male sex, age > 60 years, graft diameter > 9 mm, a bacterial or herpetic etiologic agent and the presence of systemic diseases such as rheumatic disorders or cancer^12^. In their series, 78% of the epithelial defects healed within 1 month.

Overall, in the present series the retransplantation rate was 15% within the first post-OP year. However, despite the severe preoperative ocular surface inflammation of the included eyes, we had only two cases (10%) of rejection during the entire follow-up period. This might be related to the induction of keratocyte apoptosis caused by CXL, thereby reducing the immunogenicity of the graft itself^13,14^. Of course, given the small sample size and retrospective nature of the study, this finding needs to be confirmed by further controlled studies. Also, this finding highlights the importance of postoperative immunologic suppression with topical corticosteroids. Even though many ophthalmologists use systemic steroids during the postoperative period in high risk cases, the quality of available evidence is uncertain, with a review from 2020 stating that no study has shown the advantage of this practice^15^. Also, some authors suggest the use of systemic cyclosporin A or mycophenolate mofetil in eyes at high risk of rejection, with the latter showing less side effects than the former^16^. However, we did not use systemic immune-suppressive medications in our patients and the analysis of their outcomes is beyond the scope of this paper.

Compared to our results, the literature shows that overall high risk corneal transplants (i.e., vascularization of the recipient, retransplantations, large graft diameters) develop rejection episodes in up to 60% of cases^17^, and eyes undergoing therapeutic keratoplasties with an active corneal infection have survival rates as low as 40% after 5 years^2,18,19^.

Only one patient in our series had atopic dermatitis and even though he developed a PED right after transplantation, this was not considered a case of sclerokeratitis since the eye was not severely inflamed^20,21^.

The main limitations of this study are the absence of a control group, small sample size, and heterogeneity of underlying diseases. The small sample size and wide range of values regarding PED after CXL-enhanced grafting make it impossible to deduct a significant improvement in the postoperative epithelial healing response. However, a trend towards shortening the time of epithelial closure was observed. We also did not consider the preoperative presence and extent of corneal neovascularization. Indeed this feature increases the risk of graft rejection and could be managed with different approaches prior to performing corneal transplantation^22^. As some studies suggest, CXL of the recipient bed is a promising approach to reduce pathologic corneal neovascularization^23^. However, at the time of patient recruitment this technique was still not sufficiently established as safe, especially given the already compromised limbal stem cells, therefore no CXL of the recipients’ peripheral bed was performed.

In conclusion, due to the severity of the included patients’ ocular histories a high rate of postoperative complications was detected, however, CXL of the graft at the time of keratoplasty decreased the need for retransplantations in eyes suffering from severe CM. Future larger prospective controlled trials are needed to further investigate the advantages of this approach.

## Data Availability

The data upon the study was based are available upon reasonable request to the corresponding author (JL).
